# A vitamin D receptor agonist converts CD4+ T cells to Foxp3+ regulatory T cells in patients with ulcerative colitis

**DOI:** 10.18632/oncotarget.18614

**Published:** 2017-06-27

**Authors:** Dong Lu, Bin Lan, Zonren Din, Hang Chen, Guoqiang Chen

**Affiliations:** ^1^ Department of Gastroenterology, The First Affiliated Hospital, Fujian Medical University, Fuzhou 350005, China; ^2^ Department of Gastroenterological Surgery, The First Affiliated Hospital, Fujian Medical University, Fuzhou 350005, China

**Keywords:** intestine, ulcerative colitis, Th2 polarization, vitamin D receptor, agonist

## Abstract

One of the pathological features of ulcerative colitis (UC) is the dysfunction of immune regulatory T cells (Treg cells); the pathogenesis is unclear and needs to be further investigated. Vitamin D has immune regulatory functions. This study tests a hypothesis that vitamin D receptor (VDR) regulates Treg cell differentiation. Peripheral blood samples were collected from UC patients and healthy subjects. The correlation between VDR expression and T helper (Th)2 cell differentiation in peripheral CD4^+^ T cells was analyzed. We observed that the expression of VDR was lower, the expression of interleukin (IL)-4 was higher, in peripheral CD4^+^ T cells of UC patients than that in healthy controls. Naive CD4^+^ T cells from VDR deficient mice were prone to differentiating into Th2 cells, which could be adjusted by the presence of VDR agonists. The Th2 polarization status in the peripheral CD4^+^ T cells of UC patients could be converted to regulatory T cells in the culture in the presence of VDR agonists. In conclusion, the peripheral Th2 cells in UC patients can be converted to regulatory T cells by VDR agonists in the culture. The results suggest that administration of VDR agonists at proper dosages may improve the immunity of UC patients.

## INTRODUCTION

Ulcerative colitis (UC) is one the forms of inflammatory bowel disease (IBD). Another form of IBD is Crohn’s disease. UC is a chronic inflammatory disease in the colon mucosa. The causative factors of UC are not fully understood yet. It is proposed that abnormal immune responses to the commensal bacteria are one of the factors to initiate inflammation in the colon mucosa [1]. Both T helper (Th)1 and Th2 types of the inflammation in the colon mucosa have been reported in UC patients [2]. The Th1 type inflammation is featured by high frequency of Th1 cells and overproduction of Th1 cytokines. The Th2 type inflammation is featured by high frequency of Th2 cells and overproduction of Th2 cytokines in the body [3]. Although research in the area of IBD advanced rapidly in the recent years, yet, the mechanism by which initiation of the Th1 polarization or Th2 polarization is still obscure. The therapeutic efficacy in Th1 or Th2 type inflammation, such as UC, is limited currently [4].

It is reported that vitamin D (VitD)-deficiency or insufficiency is associated with the pathogenesis of IBD [5]. VitD is one of the fat-soluble vitamins responsible for increasing absorption of calcium, iron, magnesium, phosphate, and zinc from the intestine. Although VitD can be absorbed from foods, few foods contain VitD; thus, the major sources of VitD are the dermal synthesis from cholesterol, which is dependent of sun exposure [6]. The VitD receptors (VDR) mediate the effects of VitD in the cell. Thus, it is reported that the insufficient expression of VDR is associated with the pathogenesis of a number of diseases, including IBD [7, 8]. Deficiency of gut epithelial VDR affects compromises gut epithelial barrier integrity [9], gut microbial assemblage and enhances susceptibility to colitis induced by dextran sulfate sodium [10]. Using VitD analogue KH1060 or BXL-62 can antagonize the effects of inflammatory mediators of IBD [11, 12]. Yet, the underlying mechanism by which the VDR deficiency or insufficiency affects the intestinal mucosa is not fully understood.

The abnormality of regulatory T cells (Treg) is also found in patients with IBD [13]. One of the functions of Treg is to suppress the immune response of other immune cells [14]. The over production of the cytokines of CD4^+^ T cells, including interleukin (IL)-1β, IL-4, IL-17, interferon-γ and tumor necrosis factor, in IBD patients suggest the abnormality of Tregs [15]. Yet, the remedies used to modulate the aberrant cytokine expression in IBD are limited at the time being. Published data indicate that the Th2 cells in subjects with immune disorders can be converted to Tregs [16]. Vitamin D can promote the generation of Treg [17]. Based on the above information, we hypothesize that promoting VDR may convert Th2 cells from IBD patients to Treg. The results of the present study showed that exposure to VDR agonists could increase the expression of VDR in Th2 cells and further converted Th2 cells to Tregs.

## RESULTS

### Lower expression of VDR in peripheral CD4^+^ T cells is correlated with serum IL-4 in UC patients

**Figure 1:**
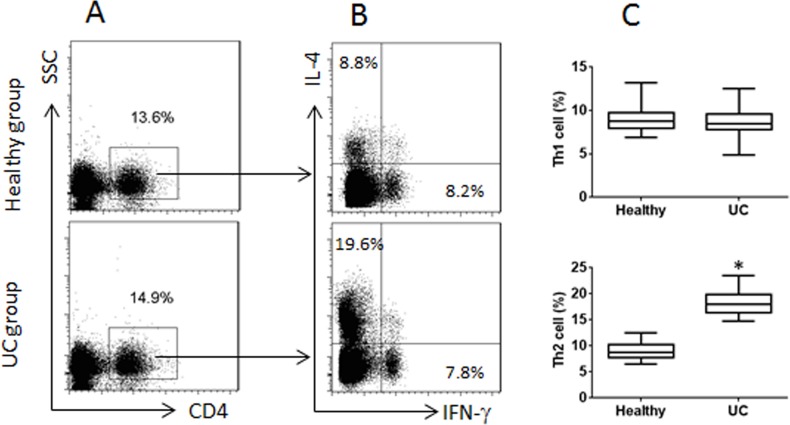
Peripheral CD4^+^ T cells in UC patients and healthy subjects Blood samples were collected from 20 UC patients and 20 healthy subjects. PBMC were isolated from the blood samples and analyzed by flow cytometry. **(A)** CD4^+^ T cells were gated. **(B)** the IL-4^+^ T cells and IFN-γ^+^ T cells were gated from the gated CD4^+^ T cells in panel A. **(C)** the floating bars indicate the summarized data [mean ± SD; *p<0.01 (t test), compared with the healthy group] of Th1 and Th2 cells in panel B. Samples from individual subjects were analyzed separately. Each experiment was repeated 3 times.

**Figure 2:**
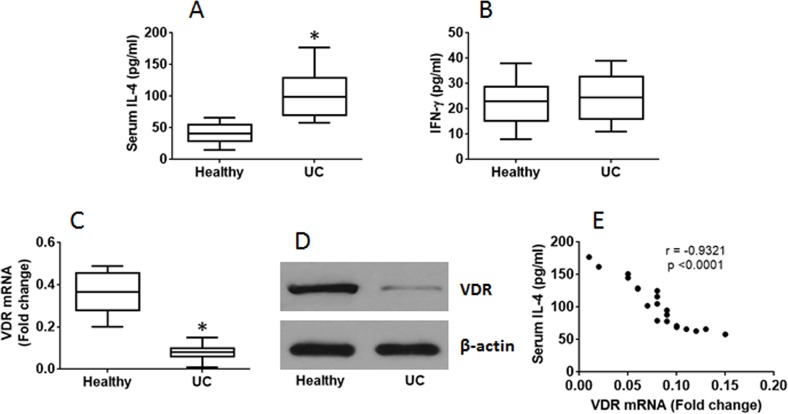
Serum IL-4 is negatively correlated with expression of VDR in CD4^+^ T cells The blood samples and PBMC were the same as that in Figure 1. **(A-B)** the results of ELISA show the levels of serum IL-4 (A) and IFN-γ (B). **(C-D)** the RT-qPCR (C) and Western blotting (D) results show VDR expression in peripheral CD4^+^ T cells. **(E)** the correlationship between the serum IL-4 and VDR mRNA in the CD4^+^ T cells of UC patients. The data of bars are mean ± SD. *p<0.01 (t test), compared with the healthy group. Samples from individual subjects were analyzed separately.

It is reported that VDR is involved in regulating immune responses [18]. To investigate if VDR expression by T cells is involved in the CD4^+^ T cell response in UC patients, we collected peripheral blood samples from 20 UC patients; the peripheral blood mononuclear cells (PBMC) were analyzed by flow cytometry. The results showed that higher frequency of CD4^+^ IL-4^+^ T cells was found in the samples from UC patients as compared with healthy controls, while the frequency of CD4^+^ IFN-γ^+^ T cells was not significantly different between UC patients and healthy controls (Figure 1). The results of serum cytokines of IL-4 and IFN-γ were in parallel to the flow cytometry results (Figure 2A-2B). On the other hand, we isolated CD4^+^ T cells from the PBMC and analyzed by RT-qPCR and Western blotting. The results showed that lower expression of VDR was detected in the CD4^+^ T cells of UC patients than that in healthy controls (Figure 2C-2D). Considering there might be a link between the expression of VDR and the Th2 polarization, we performed a correlation test with the data. The results revealed a negative correlation between the expression of VDR in CD4^+^ T cells and the serum levels of IL-4 in UC patients (Figure 2E). The results indicate that UC patients have the Th2 polarization status in the peripheral blood system, which is negatively correlated with the expression of VDR in the peripheral CD4^+^ T cells.

### VDR^−/−^ naive CD4^+^ T cells are prone to differentiating into Th2 cells

**Figure 3:**
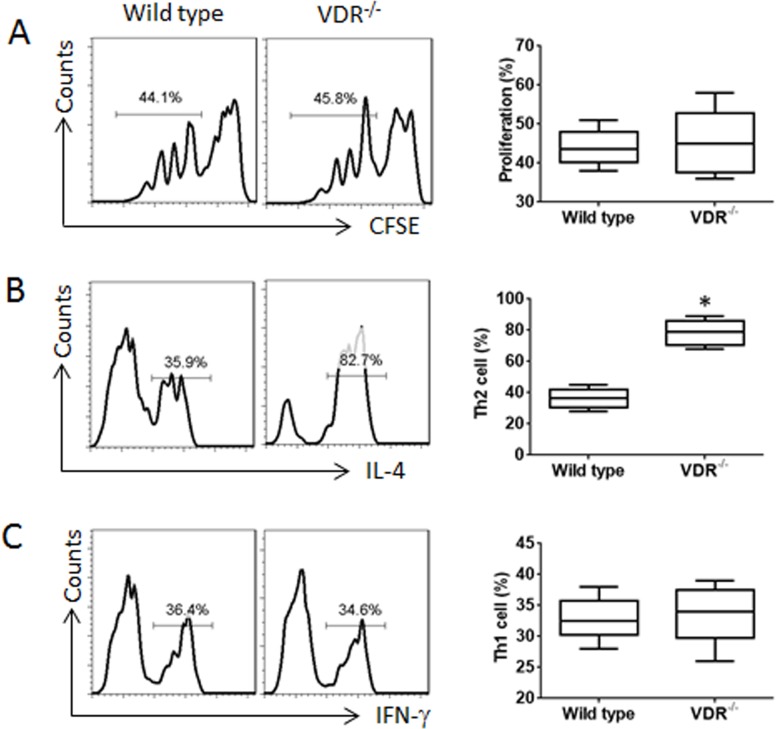
VDR^−/−^ CD4^+^ T cells are prone to differentiating into Th2 cells **(A)** CFSE-dilution assay results, of which naive CD4^+^ T cells were labeled with CFSE and cultured for 3 days in the presence of the activating reagents, including antibodies of CD3 (coated the culturing wells) and CD28 (5 μg/ml), PMA (10 ng/ml) and ionomycin (100 ng/ml). **(B-C)** the flow cytometry histograms show the frequency of Th2 cells (B) and Th1 cells (C) after the naive CD4^+^ T cells were cultured for 6 days in the presence of the activating reagents. Brefeldin A (10 μg/ml) was added to the culture in the last 3 h. The floating bar graphs show the summarized flow cytometry histogram data on the left side. The data of bars are presented as mean ± SD; *p<0.01 (t test), compared with the wild type mice. The data are summarized from 3 independent experiments.

The data of Figure 2 implicate that VDR in T cells may be involved in the lineage differentiation. To test this, we isolated naive T cells (CD4^+^ CD25^−^ CD62L^+^) from VDR^−/−^ mouse and the wild type mouse spleen. The cells were labeled with CFSE and stimulated with anti-CD3/CD28 antibodies and PMA/ionomycin in the culture for 3 days and analyzed by flow cytometry. The results showed that the naive T cells from both VDR^−/−^ mice and the wild type mice proliferated in response to the stimulation (Figure 3A). In separate experiments, naive CD4^+^ T cells were cultured for 6 days in the presence of anti-CD3/CD28 antibodies and PMA/ionomycin. The cells were analyzed by flow cytometry. The results showed much more Th2 cells in VDR^−/−^ CD4^+^ T cells than that in the wild type CD4^+^ T cells, while the frequency of Th1 cells was not significantly different from both groups (Figure 3B-3C). The results indicate that the VDR^−/−^ CD4^+^ T cells are prone to differentiating Th2 cells.

### VDR suppresses Th2 cells via modulating the gene transcription

**Figure 4:**
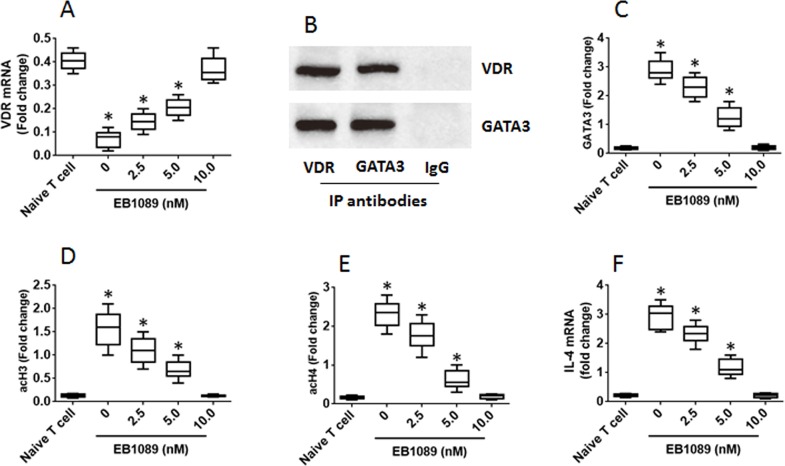
VDR agonists down regulate IL-4 gene transcription in Th2 cells Th2 cells were generated from mouse spleen cells and cultured for 3 days in the presence of EB1089 (a VDR agonist) at the indicated concentrations as denoted on the X axis. **(A)** the VDR mRNA levels. **(B)** the complex of VDR and GATA3. **(C-E)** the levels of GATA3, acH3 and acH4 at the IL-4 promoter locus. **(F)** the levels of IL-14 mRNA. The data of bars are presented as mean ± SD. *p<0.01 (t test), compared with the naive T cells. The data represent 3 independent experiments.

The data of Figure 3 and implicate that VDR may be associated with the homeostasis of the Th1/Th2 balance programme in CD4^+^ T cells. To test this, we prepared Th2 cells first, then stimulated the Th2 cells with EB1089 (a VDR agonist), which up regulated the expression of VDR in the T cells in a dose-dependent manner (Figure 4A). We also detected a complex of VDR and GATA3 in the Th2 cells (Figure 4B), from which we inferred that such a physical contact between VDR and GATA3 might interfere with the function of GATA3. Indeed, we observed that the GATA3 levels at the IL-4 promoter locus were down regulated by the exposure to VDR agonists in the culture (Figure 4C); the levels of acetylated histone 3 and histone 4 were also decreased upon exposing to the VDR agonist (Figure 4D-4E). Finally, we assessed the expression of IL-4 in the Th2 cells. The results showed that exposure to EB1089 down regulated the expression in a dose-dependent manner (Figure 4F). The results demonstrate that the expression of VDR in Th2 cells can be enhanced by VDR agonists, following which the expression of IL-4 can be down regulated.

### VDR agonists convert Th2 cells to CD4^+^ CD25^+^ Foxp3^+^ Treg

**Figure 5:**
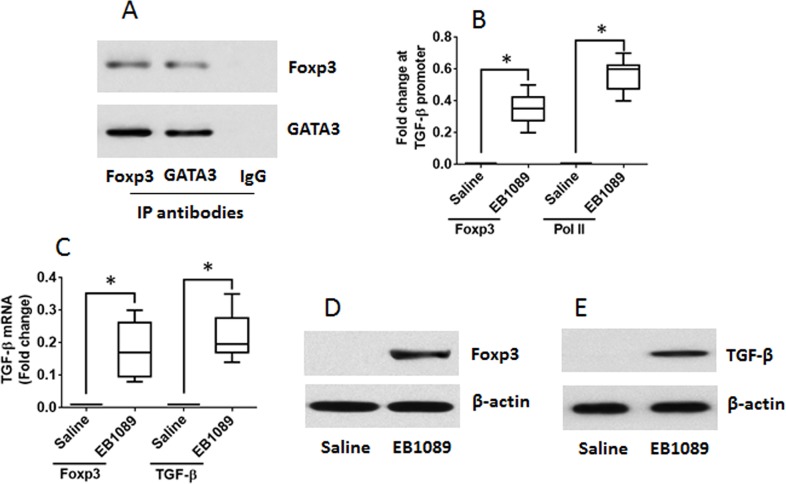
VDR agonists convert Th2 cells to Tregs Th2 cells were generated from mouse spleen cells. **(A)** IP results show a complex of GATA3 and Foxp3 was detected in the Th2 cells. **(B-E)** the Th2 cells were cultured for 6 days in the presence of EB1089 (10 nM) and T cell activators. **(B)** ChIP results show the binding of Foxp3 to and the Pol II levels at the TGF-β promoter locus. **(C)** RT-qPCR results show the levels of Foxp3 and TGF-β in the T cells. **(D-E)** Western blotting results show the protein levels of Foxp3 and TGF-β in the T cells. The data of bars are presented as mean ± SD. *p<0.01, compared with the saline group. The data represent 3 independent experiments.

**Figure 6:**
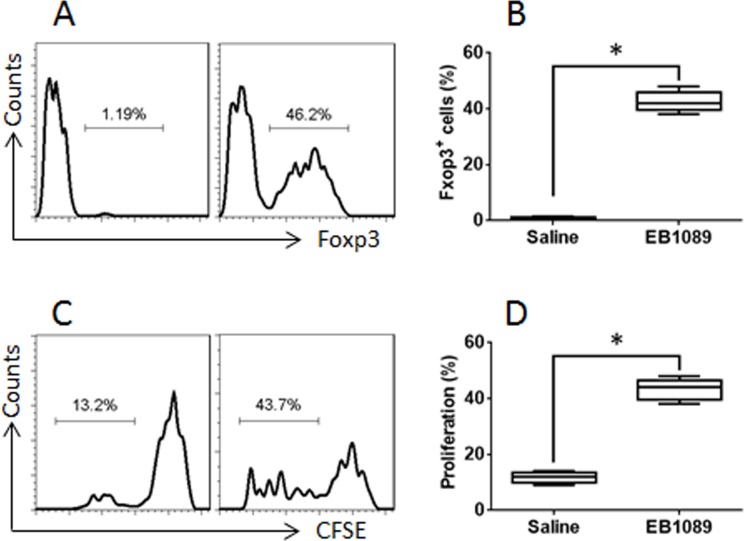
VDR agonists convert UC patients’ CD4^+^ T cells to Tregs Blood samples were collected from 6 UC patients. The CD4^+^ CD25^+^ CD127^+^ T cells were isolated from the PBMC by MACS and cultured with EB1089 (10 nM) for 6 days. **(A)** flow cytometry results show the frequency of Foxp3^+^ cells converted by EB1089. **(B)** the summarized data of panel A. **(C)** the CD4^+^ CD25^+^ CD127^−^ Tregs were isolated from the T cells of panel A by MACS. Effector T cells (Teff; CD4^+^ CD25^−^, labeled with CFSE) were isolated from healthy subjects by MACS. The flow cytometry results show the proliferation of the Teff cells. **(D)** the summarized data of panel C. The data of bars are presented as mean ± SD. *p<0.01, compared with the saline group. The data represent 3 independent experiments.

Published data indicate that GATA3 binds to Foxp3 to form a complex to interfere with the function of Foxp3 [19]. Thus, we assumed that after binding to VDR, GATA3 might release the binding to Foxp3. Indeed, a complex of GATA3 and Foxp3 was identified in Th2 cells (Figure 5A). As shown by ChIP assay, there was almost no Foxp3 binding to the promoter of TGF-β in Th2 cells. After treating with EB1089, however, the levels of Foxp3 were significantly increased at the promoter locus of TGF-β, at where the Pol II levels were also increased (Figure 5B). This phenomenon implicates that the expression of TGF-β might be up regulated in the cells. Therefore, we analyzed the cells with RT-qPCR and Western blotting. Indeed, the expression of Foxp3 and TGF-β was significantly up regulated (Figure 5C-5E). The results demonstrate that exposure to VDR agonists can convert Th2 cells to Tregs. To elucidate if the converted Tregs have immune suppressive functions, we cocultured naive CD4^+^ T cells and the converted Tregs for 3 days in the presence of the T cell activators. The results showed that the converted Tregs significantly suppressed the CD4^+^ T cell proliferation. To elucidate if VDR agonists also convert CD4^+^ T cells of UC patients to Tregs, we collected blood samples from UC patients; the CD4^+^ CD25^+^ CD127^+^ T cells were isolated from PBMC and cultured for 6 days in the presence of EB1089. As shown by flow cytometry data, more than 40% CD4^+^ T cells were converted into Foxp3^+^ Tregs (Figure 6A-6B). The CD4^+^ CD25^+^ CD127^−^ Tregs were isolated by MACS and cultured with CD4^+^ CD25^−^ naive T cells (the effector T cells; from healthy subjects, labeled with CFSE) for 3 days in the presence of T cell activators. As shown by flow cytometry data, the converted Tregs significantly suppressed the effector T cell proliferation (Figure 6C-6D). The results demonstrate that VDR agonists can convert Th2 cells to Tregs; the latter has immune suppressive functions.

## DISCUSSION

Based on the novel finding that the abnormally higher expression of IL-4 was negatively correlated with the expression of VDR in Th2 cells of UC patients, we further found that the up regulation of VDR in Th2 cells by VDR agonists converted the Th2 cells into Tregs. The data have revealed a potential approach in the improvement of the immune response in the intestine of IBD patients by using VDR agonists that can up regulate the generation of Treg in IBD patients.

We found a previously unknown phenomenon in the present study that the Th2 cells from UC patients could be converted to Tregs. Although Th2 cells were considered as a fraction of terminal T cells, published data indicate that Th2 cells can still be converted to other cell types; such as Liu et al reported that mast cell-derived serine proteinase converted Th2 cells to Tregs via up regulating the expression of the B cell lymphoma 6 protein, the latter further inhibited the expression of GATA3 and up regulated the expression of Foxp3. In addition, others also found that Th17 cells could be converted to Tregs [20]. Our data indicate the Th2 cell’s plasticity that may be a therapeutic opportunity for inflammatory diseases with Th2 polarization.

The data show that VDR agonists not only can up regulate the expression of VDR in Th2 cells, but can also convert Th2 cells to Tregs. The purpose of up regulating the expression of VDR by VDR agonist is mainly used to inhibit inflammatory reactions; such as inhibiting peritoneal inflammation by the inhibition of macrophage recruitment and inflammatory cytokine secretion [21], inhibiting chronic inflammation and anti-cancer [22]. Studies also found that VDR agonists can regulate the aberrant immune resposnes; such as improving the allergen-triggered eczema in mice [22]; modulating B cell activities to suppress the production of IgE [23] and suppressing the Th17 cell polarization-related nephritis [24]. Our data are in line with those previous studies by showing that VDR agonists can regulate Th2 cell properties from UC patients.

The therapeutic effects of UC are not satisfactory currently. Using anti-TNF antibodies can ameliorate the clinical symptoms in some of the UC patients [25]. Steroids are the common and effective therapeutic for IBD patients [26]; but the severe side effects limit their use [27]. To up regulate the generation of Treg has been tried in IBD both in animal models and IBD patients [28, 29]. Our data have revealed a novel aspect in this study area that administration of VDR agonists also up regulates the generation of Treg by converting Th2 cells to Foxp3^+^ Tregs.

Published data show that VitD3 can also suppress Th1 response. Such as Vidyanani et al reported that VitD3 could suppress IFN-γ and IL-12 in peripheral leukocytes from patients with Mycobacteria tuberculosis [30], while Di Filippo et al observed that administration with VitD supplements suppressed both IL-4 and IFN-γ in the serum of patients with atopic dermatitis [31]. Ozkara et al indicated that patients with polyposis/allergic rhinitis showed a negative correlation between serum VitD and IL-4, a positive correlation between serum VitD and IFN-γ. However, in a group of polyposis/non-allergic rhinitis patients, serum VitD was positively correlated with IL-4 and negatively correlated with IFN-γ levels [32]. Our data are in line with the reports of Di Filippo [31] and Ozkara [32] by showing that VDR has the effects to down regulate the expression of IL-4 or the Th2 response.

In summary, the present study has revealed that the expression of IL-4 in peripheral CD4^+^ T cells of UC patients is negatively correlated with the expression of VDR. Stimulation of peripheral CD4^+^ T cells of UC patients with VDR agonists can convert the CD4^+^ T cells to Foxp3^+^ Tregs, indicating that Th2 cells are still convertible in UC patients. Since Th2 polarization plays an important role in a fraction of UC patients, to convert Th2 cells to Tregs can be a novel approach to reconcile the pathology in UC patients.

## MATERIALS AND METHODS

### Reagents

The EB1089 was purchased from Tocris Bioscience (Bristol, UK). The antibodies of VDR (D-6), CD3 (F7.2.38), CD28 (CD28.2), GATA3 (HG3-31), acH3 (AH3-120), acH4 (B-10), Foxp3 (2A11G9) and TGF-β (C-16) were purchased from Santa Cruz Biotech (Santa Cruz, CA). The fluorescence labeled antibodies for flow cytometry (flow cytometry) were purchased from BD Bioscience (Franklin Lakes, NJ). The ELISA kits of IL-4 and IFN-γ were purchased from R&D Systems (Minneapolis, MN). The immune cell isolation reagent kits were purchased from Miltenyi Biotech (San Diego, CA). The reagents and materials for RT-qPCR and Western blot were purchased from Invitrogen (Carlsbad, CA). The cocktail of phorbol 12-myristate 13-acetate (PMA) and ionomycin, reagents for IP (immune precipitation) and ChIP (cromatin IP) were purchased from Sigma Aldrich (St. Louis., MO).

### Human subjects

UC patients were recruited into this study at our IBD clinic in the period of October 2015 to September 2016. The diagnosis and treatment of UC were carried out by our doctors. The demographic data of the patients are presented in Table 1. All the UC patients recruited in the present study were at remission period. Patients with one of the following conditions were excluded: Using immune suppressors; suffering from cancer or other immune diseases or severe organ diseases. In addition, 20 healthy subjects were also recuited into this study as controls (Table 1). The using human tissue in the present study was approved by the Human Ethics Committee at Fujian Medical University. An informed written consent was obtained from each human subject.

**Table 1:** Demographic data

Characteristic	UC	Healthy
	(N = 20)	(N = 20)
Age-year	33.6 ± 5.2	31.2 ± 2.6
Male-No. (%)	10 (50.0)	10 (50.0)
Body weight-kg	58.3 ± 6.8	62.3 ± 5.6
Current smoker-No. (%)	2 (10.0)	2 (10.0)
Duration of disease-year	4.1 ± 2.3	
Fecal calprotectin-mg/g	1.1 ± 0.9	
**Site of disease-No. (%)**		
Left side of colon	6 (30.0)	
Proximal colon	6 (30.0)	
All of the colon	8 (40.0)	
**Fecal calprotectin**	248.6 ± 43.5	
**Hemoglobin-g/L**	125.5± 11.5	
**WBC** (× 10^−9^/L)	8.7± 2.8	

### Blood sample collection

Peripheral blood samples were collected from each human subject (40 ml/person) by ulnar vein puncture. The peripheral blood mononuclear cells were isolated from the blood samples by gradient density centrifugation.

### Cell culture

The PBMC were cultured in RPMI1640 medium supplemented with 2 mM glutamine, 10% fetal bovine serum, 0.1 mg/ml streptomycin and 100 U/ml penicillin. The medium was changed in 2-3 days. The cell viability was greater than 99% as assessed by Trypan blue exclusion assay.

### Immune cell isolation

The immune cells used in the experiments were isolated by magnetic cells sorting (MACS) with purchased reagent kits following the manufacturer’s instructions. The cell purity was greater than 96% as assessed by flow cytometry.

### Flow cytometry (flow cytometry)

The cells were collected from related experiments. In the surface staining, the cells were stained with fluororescence-labeled antibodies of interest or isotype IgG for 30 min at 4°C. If the intracellular staining was required, the cells were fixed and permealized by incubating with 1% paraformaldehyde for 30 min and incubating with 0.5% saponin. The cells were then stained with fluororescence-labeled antibodies of interest or isotype IgG for 30 min at 4°C. After washing, the cells were analyzed with a flow cytometry device (FACSCanto II, BD Bioscience). The data were analyzed with software flowjo. The data of isotype IgG were used as a gating reference.

### Emzyme-linked immunosorbent assay (ELISA)

Cytokines in the serum were determined by ELISA with purchased reagent kits following the manufacturer’s instructions.

### Real time quantitative RT-PCR (RT-qPCR)

Total RNA was extracted from cells collected from related experiments and converted to cDNA with a reverse transcription kit following the manufacturer’s instructions. The samples were then amplified in a qPCR device (CFX96^TM^, Bio-Rad) with the SYBR Green Master Mix. The results were normalized to fold change against the housekeeping gene β-actin. The primers used in the present study are presented in Table 2.

**Table 2:** Primers used in the present study

Molecules	Forward	Reverse
VDR	tatgacctgtgaaggctgca	atcatctcccgcttcctctg
IL-4	aacgaggtcacaggagaagg	tctgcagctccatgagaaca
TGF-β	cctgcaagaccatcgacatg	tgttgtacaaagcgagcacc
TGF-β promoter (−533 to −15)	tctcatgggtaaggtgcctc	tgggagttgttgaagggtca
*Il4* promoter (−688 to −32)	ggcctctcccttctatgcaa	gattgtcagtcacttggggc

### Western blot

Total proteins were extracted from cells collected from related experiments. The proteins (80 μg/well) were fractioned by SDS-PAGE (sodium dodecyl sulfate polyacrylamide gel electrophoresis) and transferred onto a PVDF membrane. The membrane was blocked by 5% skim milk for 30 min, incubated with the primery antibodies of interest or isotype IgG (negative controls) overnight at 4°C, washed with TBST (Tris-buffered saline Tween 20), incubated with peroxidase-labeled second antibodies for 1 h at room temperature and washed with TBST. The blots on the membrane were developed with ECL (enhanced chemiluminescence) and photographed with an imaging device (UVI, Cambridge, UK).

### Mice

The VDR−/− mice and the littermate C57BL/6 mice (8-10 week old) were purchased from Beijing Experimental Animal Center (Beijing, China). The mice were maintained in a specific pathogen-free facility at Fujian Medical University. The experimental procedures were approved by the Animal Ethics Committee at Fujian Medical University.

### T cell proliferation assay

CD4^+^ CD25^−^ T cells (effector T cells; Teff) and CD4^+^ CD25^+^ CD127^−^ T cells (Treg) were isolated by MACS with purchased reagent kits following the manufacturer’s instructions. The purity of the isolated cells was greater than 96% as assessed by flow cytometry. The Teffs (labeled with CFSE) were cultured with Tregs for 3 days in the presence of T cell activators [including antibodies of CD3 (coated the culturing wells) and CD28 (5 μg/ml), PMA (10 ng/ml) and ionomycin (100 ng/ml)]. The cells were analyzed by flow cytometry (the CFSE-dilution assay). The results are presented as the percentage of proliferating Teffs against the total Teffs.

### Generation of Th2 cells

CD4^+^ CD25^−^ T cells were isolated from spleen cells by MACS using commercial reagent kits following the manufacturer’s instruction. The cells were cultured in the presence of the T cell activators (see above) and anti-IFN-γ antibody (100 pg/ml) for 6 days. The CD4^+^ CD25^+^ CD127^+^ T cells were isolated from the cells by MACS. As assessed by flow cytometry, the IL-4^+^ T cells (Th2 cells) were greater than 96%. The cells were used as Th2 cells in the following experiments.

### Immunoprecipitation assay (IP)

The cell lysates were prepared with cells collected from related experiments and precleared by incubating with protein G agarose at 4°C for 2 h. The supernatant was collected and incubated with antibodies of interest of isotype IgG overnight at 4°C to form immune complexes. The immune complexes were precipitated by incubating with protein G agarose at 4°C for 2 h. The proteins on the beads were eluted with an eluting buffer and analyzed by Western blotting.

### Chromatin IP (ChIP)

Cells collected from related experiments were fixed with 1% formalin for 15 min to cross-link the chromatin and the proteins. The cells were lysed with a lysing buffer followed by sonicating to shear the chromatin into small pieces (200-500 bps). The samples were then processed by the IP procedures. The eluted samples were processed to recover the DNA. The DNA was then analyzed by qPCR. The primers of the TGF-β promoter used in the present study are presented in Table 2. The results are presented as fold change against the input.

### Statistics

The difference between two groups was determined by Student t test or ANOVA if more than two groups. The correlation between two groups was analyzed by the Pearson correlation test. P<0.05 was set as a significant criterion.
